# Seasonal Prevalence of Helminthic Infections in the Gastrointestinal Tract of Sheep in Mazandaran Province, Northern Iran

**DOI:** 10.1155/2022/7392801

**Published:** 2022-12-21

**Authors:** Alireza Salehi, Mahsa Razavi, Nasrollah Vahedi Nouri

**Affiliations:** ^1^Department of Veterinary Parasitology, Islamic Azad University, Babol Branch, Mazandaran, Iran; ^2^Department of Animal Parasitic Disease Research, Razi Vaccine and Serum Research Institute, Agricultural Research, Education and Extension Organization, Karaj, Iran

## Abstract

Helminthic infection is the major cause of the sheep's reduced productivity. In this study, a total number of 240 fecal samples of sheep from stationary flocks of four different zones of Mazandaran province (Amol, Babol, Sari, and Nowshahr cities) were examined each season, out of which 53.33% of animals were affected by the helminthic infections. The most prevalent infecting parasites were the *Trichostrongylidae* (46.61%), followed by the *Fasciola* (9.96%). In addition, the *Strongyloides* had the lowest proportion with only 2.39%. The other detected parasites included *Chabertia* (5.98%), *Cooperia* (3.19%), *Nematodirus* (3.19%), *Trichuris* (5.58%), *Toxocaridae* (4.78%), *Haemonchus* (4.78%), *Ostertagia* (5.58%), *Oesophagostomum* (4.78%), and *Dicrocoelium* (3.19%). The nematodes had the highest percentage with 86.85%, whereas the trematodes followed them with 13.15%. No significant difference was observed between the infection level in females and males, with 56.9% and 43.94% rates of infection, respectively. Significantly lower infection was observed in winter compared with the other seasons. *Trichostrongylidae* was the dominant genus across all seasons. It was also noted that winter had the lowest percentage of helminthic infection significantly. The eggs per gram of feces was also estimated, and it showed that a significant number of sheep are infected with a moderate number of parasites. In a conclusion, even though livestock farmers have been using antiparasitic drugs in their livestock in recent years, parasitic infection still exists in livestock. Thus, a proper implementation of helminthic infection control programs in this area should be employed as the key element for reducing the high prevalence of livestock helminthic infection.

## 1. Introduction

Sheep husbandry has been popular in Northern Iran for a long time, and this animal plays a substantial role in the economy of this region. The majority of people consider lamb to be the best red meat because of its exceptionally soft texture and mouthwatering flavor. One of the top issues facing a rancher today is infectious illnesses in sheep. Parasitic infections, particularly gastrointestinal helminthic worms in tropical and subtropical areas, are of paramount concern to them [1]. The helminth infections are the major barrier to the sheep's productivity [2]. Economic losses from gastrointestinal parasites include mortality, weight loss, and a decreased output of both meat and milk [3].

The migration of parasitic larvae upon intake is one of the most serious side effects of gastrointestinal parasitic diseases [4]. Rough skin, weakness, diarrhea, restlessness, hypoproteinemia, edema of the mandible (bottle jaw), loss of appetite, and weight loss are the symptoms that an infected sheep exhibits. For example, the *Trichostrongylus* nematodes can destroy the proteins and red blood cells, which can lead to anemia, and cause the animals to lose weight and become weak [5].

The occurrence of gastrointestinal helminthic infections is highly affected by a combination of risk factors, including ecological conditions, animal husbandry, antiparasitic consumption interval, pasture management, host species, animal gender, age, season, body condition, and race [6]. The *Haemonchus*, *Trichostrongylus*, *Ostertagia*, and *Nematodirus* genera are the most prevalent gastrointestinal nematodes in small ruminants worldwide [2, 7]. Research on the parasitic infections of small ruminants' gastrointestinal tracts in various Iranian regions has been conducted [8–11].

Mazandaran is located at 36.23°N, 52.53°E, and covers an area of 23,833 km^2^, accounting for 1.5% of Iran's total land area. With its rich grasslands, Mazandaran province in Northern Iran is one of the best regions for sheep rearing and contributes significantly to mutton production. However, sheep are exposed to wide parasites when grazing in contaminated lands [12]. The goal of the current study was to identify gastrointestinal parasites, assess the severity of infection in animals, and evaluate the risk factors for sheep in four major cities of Mazandaran province.

## 2. Materials and Methods

### 2.1. Study Area and Samples

Mazandaran is located in Northern Iran, on the Caspian Sea's southern coast. This province, which is located at 36.23°N, 52.53°E and covers an area of 23,833 km^2^, accounts for 1.5% of Iran's total land area. Like the Great Wall, the Alborz Mountain range has split Mazandaran into lowlands and mountains. Due to the sea, mountains, and forests, Mazandaran's climate is classified into two types: moderate humidity and mountainous. The presence of the Caspian Sea and the Alborz Mountains, as well as their proximity to each other, has resulted in a temperate and humid climate, with particularly hot and humid summers on the shore. Winters in these areas are moderate and humid, with only a few frosty days. For these reasons, animal husbandry is one of the most prominent occupations in this area due to the ideal geographical conditions.

The status of traditional farms in Nowshahr, Babol, Amol, and Sari cities as sampling regions of Mazandaran province was determined in the course of this research in 2020 across four seasons, which included field and laboratory studies. Out of 240 samples gathered over a year, 120 of them were male, and the rest were female. Four random herds with more than 200 sheep were chosen in each area in spring, and 15 samples were obtained from each herd randomly each season afterward. The animals' gender was recorded on the necessary forms. Sampling was done in the middle of each season. Approximately 30–50 g of excrement was collected straight from the animal's rectum and kept in the special sample collection containers individually. It should be noted that albendazole, an antiparasitic drug, was used by farmers twice a year at the beginning of spring and autumn in this region annually. To avoid the hatching of larvae eggs, the samples were evaluated in the laboratory 2 hours after sampling.

### 2.2. Parasitological Examination

The Willis method was employed in this regard [13]. Based on an earlier study [14], after gathering enough samples, approximately 3 g of excrement with 42 ml of regular water was mixed. Then, using a glass ball and sandpaper, the mixture in a glass was stirred until it was homogenous. A 100-mesh sieve was used to filter the prepared mixture in the beaker. After stirring, a portion of the filtered liquid was placed into a Clayton-Lane centrifuge tube with a volume of 15 ml. The liquid was centrifuged for 2 minutes at 1500 rpm. Water was used to perform the washing process numerous times. The upper liquid, which contained grease and colors, was discarded after washing, and the remaining sediment from the bottom of the tube was removed by flipping to the sediment region at the tube's end. Fill the tube halfway with saturated sodium chloride water solution and gently mix it with a thumb as the lid several times. After that, the tube was filled to the brim with saturated solutions and centrifuged. After that, the laminated tubes were filled to the brim with saturated solutions and centrifuged for 2 minutes at 1000 rpm. The lamellae were then placed on a slide and examined under a microscope carefully. The number of eggs counted below the lamellae represents the number of eggs per gram (EPG) of excrement since the total volume of the initial mixture is 45 ml, and the volume of the centrifuge tube is 15 ml. At this point, 1/6 of the eggs were still in the silt at the bottom of the tube; thus, the number of eggs mentioned below the lamellar must be increased. The coefficient of adjustment of fecal concentration (normal feces = 1, semi-loose = 1.5, loose = 2, and diarrhea = 3) should also be included in the calculation for the definitive determination of the number of EPG of feces. Heavy eggs, such as *Fasciola*, *Dicrocoelium*, *Amphistomes* eggs, and larvae of pulmonary worms, are found in the sediment at the bottom of the tube after this stage, as well as some light eggs and *Trichuris* eggs. The prior solution was removed first, followed by the sediment from the tube's bottom, but instead of sodium chloride alone, a saturated room-temperature solution of zinc chloride and sodium chloride was used to float the trematode eggs [15].

### 2.3. Data Analysis

Three levels of contamination were determined based on the number of EPG of fecal sample: low contamination (the average number is less than 100 EPG of fecal sample), moderate contamination (the average number is between 100 and 500EPG of fecal sample), and high contamination (the average number is greater than 500 EPG of fecal sample). The findings were analyzed using the chi-square test based. In addition, in the fecal test, the eggs of nematodes (*Haemonchus*, *Ostertagia*, *Cooperia*, *Trichostrongylus*, *Oesophagostomum*, and *Chabertia*) whose type can be difficult to differentiate from the appearance of eggs were tallied together as *strongylids*.

## 3. Result

Overall, it was observed that the small ruminants are infected substantially in Northern Iran at a total percentage of 53.33%. The infecting parasites consist of the *Nematodirus, Trichuris*, *Toxocaridae*, *Strongyloides*, *Haemonchus*, *Ostertagia*, *Cooperia*, *Trichostrongylidae*, *Oesophagostomum*, and *Chabertia*, from the nematode genera, and the *Fasciola and Dicrocoelium*, from the trematode genera.

### 3.1. Seasonal Findings

Based on the seasonal findings, a significant difference was seen between the seasons. Fall had the highest proportion of the infection at 73.33%, followed by the summer with 66.67%. Meanwhile, the lowest rate of infection was seen in the winter with only 15%, and spring was the third season with a 58.33% infection rate among sheep. The difference between the infection rate of the seasons was statistically significant (*P* < 0.05; Table 1).

### 3.2. Gender Comparison

Regarding the rate of infection between the genders, no orientation was observed, and the helminthic lesions were not directed into a specific gender. The findings showed that helminthic infection in the female sheep was not significantly different from that of the male sheep. Moreover, the average frequency of helminthic infection in females and males were 56.9% and 43.94%, respectively.

### 3.3. Types of Parasites

Throughout the study, 12 types of different parasites were isolated by their egg or larvae. The most frequent parasite was the *Trichostrongylidae*, which had the highest rate in all seasons (Figures 1, 2, 3, 4, 5, 6, 7, 8, 9, 10, and 11).

### 3.4. Frequency

The intensity of infection in sheep was calculated based on the EPG. As a result, it was observed that there was not a high infection intensity. In addition, the first three seasons had a moderate infection intensity, whereas winter had a lower infection rate compared with them (Table 2).

## 4. Discussion

According to the results obtained in this research, the average percentage of sheep with helminthic infections in the gastrointestinal tract in Mazandaran province is 53.33%. Considering that all the studied animals were using antiparasitic drugs, such as albendazole, this level of infection is significant. This result is approximately similar to the results of previous research by Admasu and Nurlign in the Northern Regions of Ethiopia [16] and Gadahi et al. in Rawalpindi and Islamabad from Pakistan [17], which, respectively, reported a 58.7% and 53.3% rate of sheep with one or more cases of gastrointestinal parasites. However, Owusu et al. from Ghana [18] and Singh et al. from Punjab, India [19] reported the infection rate of sheep with digestive worms as 94.5% and 83.03%, respectively. In addition, Awaludin and Nusantoro stated that in their study on sheep in the East Java region of Indonesia, the rate of infection with parasitic worms was determined to be 48% [20]. This difference may be due to the differences in geographical locations, weather conditions, sheep breeding, management, and diversity in sampling methods [21].

Based on the obtained results, the average percentage of gastrointestinal parasite infection in sheep in different seasons showed that the lowest level of infection occurred in the winter (15%), and its difference compared with other seasons was statistically significant. Similarly, Vahedi et al.'s research on cattle in Mazandaran province showed that the percentage of cattle helminthic infections in the winter was 18.33%, and it was less in other seasons significantly, too [14]. In addition, the research of Jithendran and Bhat on the ruminants in the Himalayan regions of India [22] and Islam et al. on sheep in the regions of Bangladesh [23] was also consistent with this issue that winter had the lowest infection rate through all seasons. Environmental factors, such as humidity, temperature, rainfall, sunlight, wind, cloud cover area, and forest cover area, play a decisive role in the epidemiology of parasitic infections in small ruminants [24]; therefore, the difference can be due to the change of uncontrollable conditions. This study was conducted in different seasons of the year. However, according to the geographical conditions of Mazandaran province, the reason for the relatively higher contamination of livestock in spring, summer, and autumn compared with winter can be two major aspects. First, due to the cold weather, the lack of access to open pastures and less grazing, and the presence of sheep in the closed area had led to a significant reduction in the infection rate of digestive parasites in sheep this season, as well as the higher management observation on livestock in the winter. Second, when the spring comes and the relative temperature and humidity conditions improve because of the rains, the rain can last long until the summer (with less intensity) until the end of the autumn season, and after that, the chances of the survival of infectious larvae and the possibility of their consumption by livestock are increased. In addition, the free grazing of animals in the pastures has increased the average percentage of parasitic infection in sheep.

In addition, based on the obtained results, the average percentage of female and male sheep with digestive parasites was determined to be 56.90% and 43.94%, respectively, and this difference was not significant (*P* < 0.05). This result is consistent with the results of previous studies [14, 23, 25, 26]. The probable cause of the existing difference can be the effect of sex hormones, which increase the level of prolactin and progesterone hormones in the blood that can be caused by stress during pregnancy or muscle weakness, and also the period of lactation that may cause a reduction of the immune defense system of the female livestock, thus, as a result, increasing the percentage of their infections [27, 28].

Overall, 12 species of gastrointestinal parasites were identified in sheep in Mazandaran province. The types of parasites that were identified in this research included gastrointestinal nematodes and trematodes. The genera of identified nematodes in sheep included *Nematodirus*, *Trichuris*, *Toxocaridae*, *Strongyloides*, *Haemonchus*, *Ostertagia*, *Cooperia*, *Trichostrongylus*, *Oesophagostomum*, and *Chabertia*, and trematodes included the genera *Fasciola* and *Dicrocoelium*. It should be noted that usually more than one parasite genus was identified from a single sheep host, which was shown before in the previous studies [29–31]. In this study, nematodes of the genera *Trichostrongylus*, *Chabertia*, and *Oesophagostomum* were among the most common parasites in sheep based on the stool culture. Unfortunately, after comparing the results of this research with those of the previous, it can be seen that the diversity of parasites in sheep of Mazandaran province is relatively high.

Based on the results of this research, in 30.48% of the studied sheep, the intensity of infection was low, and 69.52% of them had a moderate intensity of infection. This difference was statistically significant (*P* < 0.01). By other means, the comparison of the intensity of infection in different seasons of the year showed that during the three seasons of spring, summer, and autumn, when the free breeding of livestock is more frequent, the intensity of the average infection is significantly higher than that of the winter. In the winter season, since the animals are mostly kept in a closed area and are under nutritional control, the amount of infection will be lower, which was shown in our study with a significantly lower intensity.

It should be mentioned that although livestock farmers have been using antiparasitic drugs in their livestock in recent years, parasitic infection still exists in livestock. Therefore, a new instructional manual for livestock farmers in the field of correct use of antiparasitic drugs is suggested, and it should be clarified for them that knowledge of the economic losses that are caused by parasites can be modified by stopping the life cycle of the parasites in any step. In addition, weather factors, such as rainfall, humidity, and temperature play an important role in parasitic infection, and control programs must keep looking at those natural factors. As a first move, livestock should not be allowed to continuously graze a pasture, rather the rotation between pastures should be used.

## 5. Conclusion

In a conclusion, it can be said that parasitic infection still exists in the livestock of Mazandaran. Considering the high percentage of parasitic infection in sheep in Mazandaran province, this issue is arguably a sign of neglect in the implementation of parasitic infection control programs. In addition, resistance to antiparasitic drugs should not be undertaken. Therefore, it is recommended that ranchers should employ parasitic infection control programs based on seasonal changes. It is advised that veterinarians should guide the farmers to use broad-spectrum antiparasitic drugs at the beginning of each season, and monitor the herd seasonally for any infections.

## Figures and Tables

**Figure 1:**
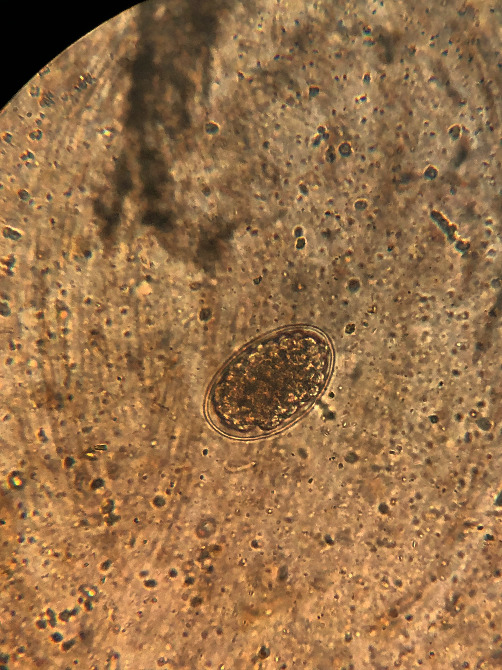
*Trichostrongylidae*: the egg is ovoid, pointed at one end, yellow-greyish in color, and thin-walled. The morula inside may consist of a variable number of blastomeres. Size: 75–95 *μ*m × 40–50 *μ*m.

**Figure 2:**
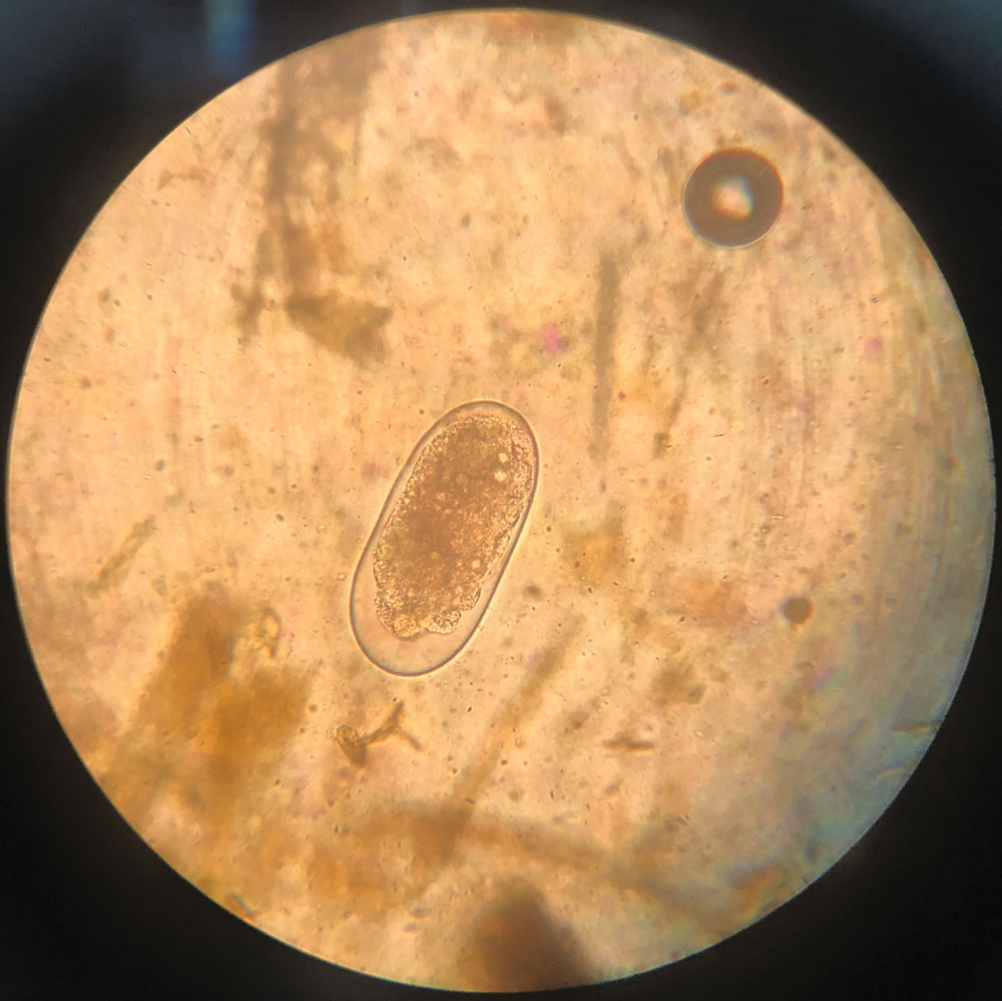
*Oesophagostomum*: the egg is ovoid, has a thin shell, measures 40–60 *μ*m × 70–100 *μ*m, and contains several cells, depending on the species.

**Figure 3:**
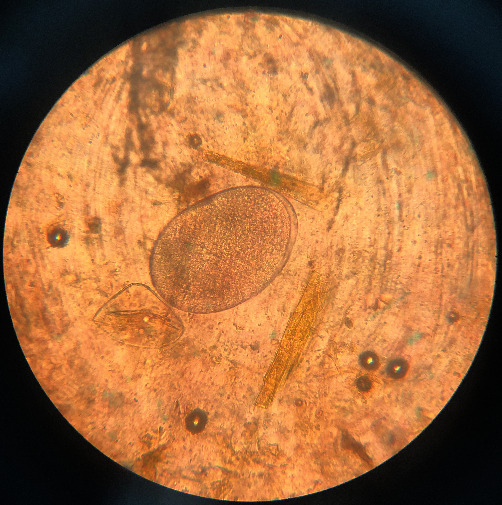
The egg of *Fasciola* sp. is large and operculated, and ovoid contains a large un-segmented ovum in a mass of yolk cells, 130–150 *μ*m × 63–90 *μ*m, light brown to yellow.

**Figure 4:**
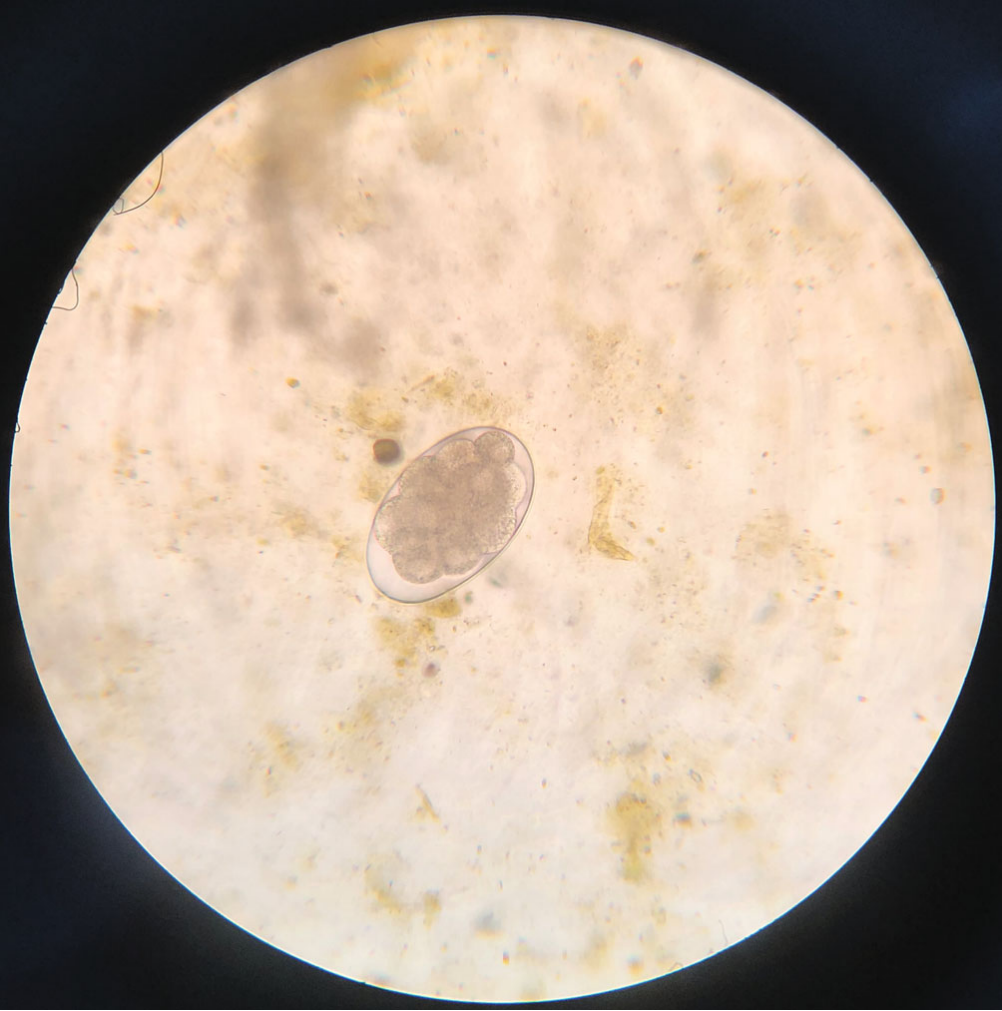
*Nematodirus*: the egg is ovoid, colorless, and thin-shelled, measuring approximately 210 *μ*m × 100 *μ*m.

**Figure 5:**
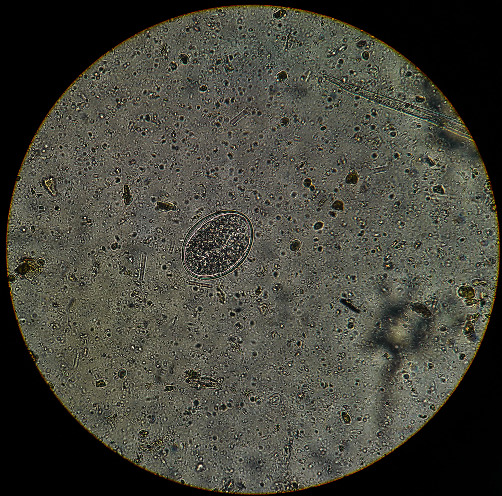
*Strongyloides*: egg of parasite. Ellipsoid, 40–85 *μ*m in length, with a thin wall containing the first-stage larva.

**Figure 6:**
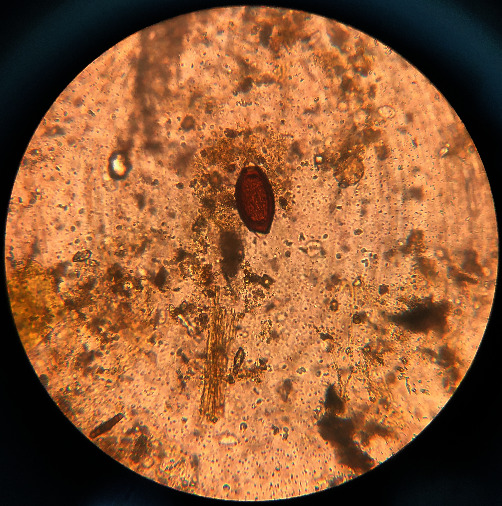
*Trichuris*: egg of parasite. Barrel-shaped eggs, 70–90 *μ*m in length, with bipolar plugs, granulation of the content.

**Figure 7:**
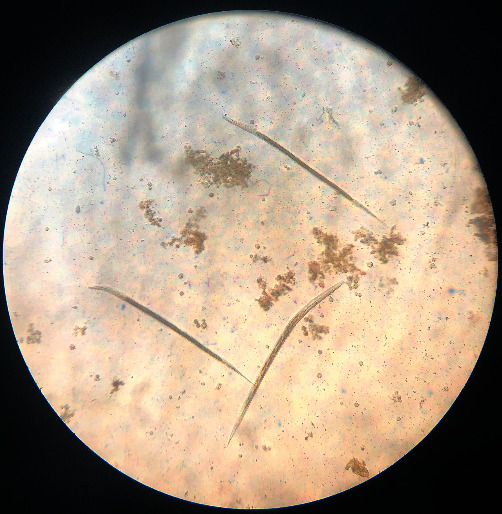
*Chabertia* sp. (tail of sheath very long, larva of medium size, 24–32 square gut cells, and lumen of gut straight). Third-stage larvae.

**Figure 8:**
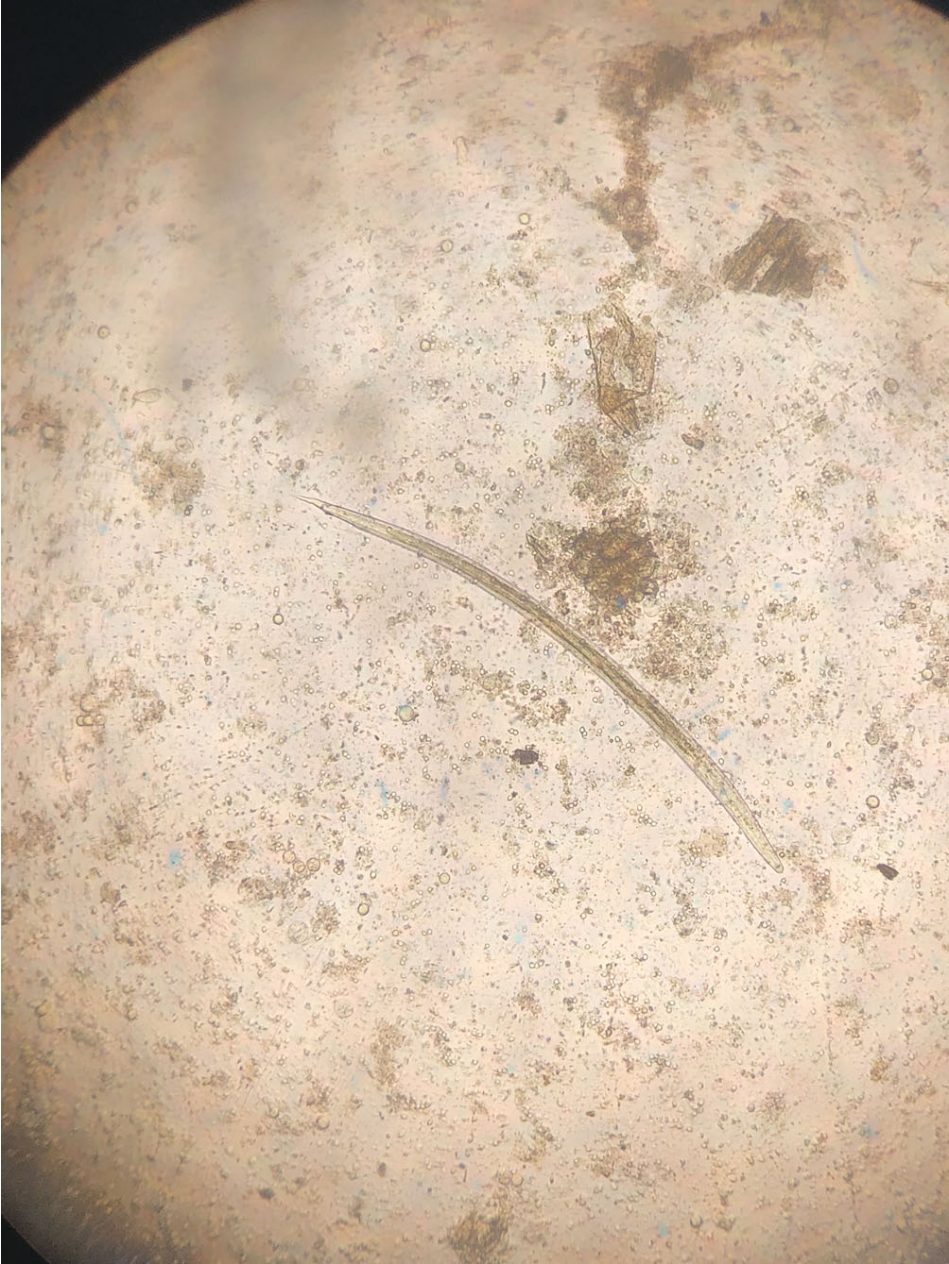
*Trichostrongylus* sp. (head rounded, short-sized larva, the tail of sheath is short, and bearing one or two tuberosities). Third-stage larvae.

**Figure 9:**
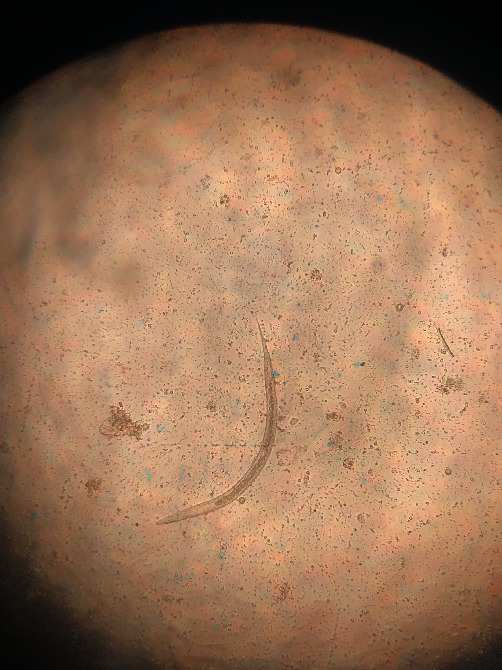
*Oesophagostomum* sp. (rounded head, medium-sized larva, long thin sheath tail, and 16–24 triangular intestinal cells). Third-stage larvae.

**Figure 10:**
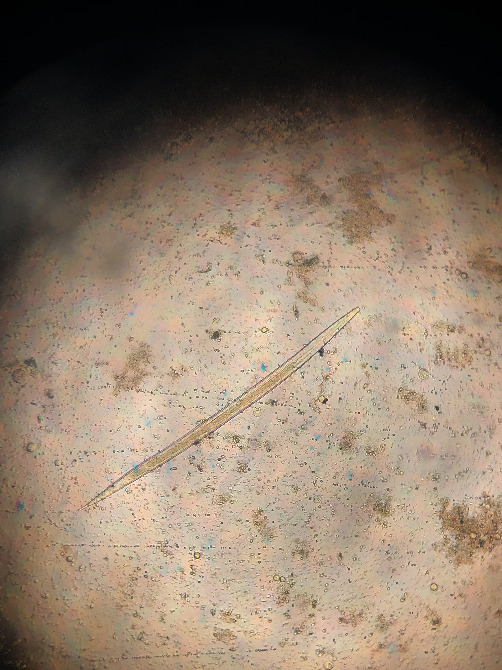
*Haemonchus* sp. (head rounded, medium-sized larva with medium length, and kinked sheath tail). Third-stage larvae.

**Figure 11:**
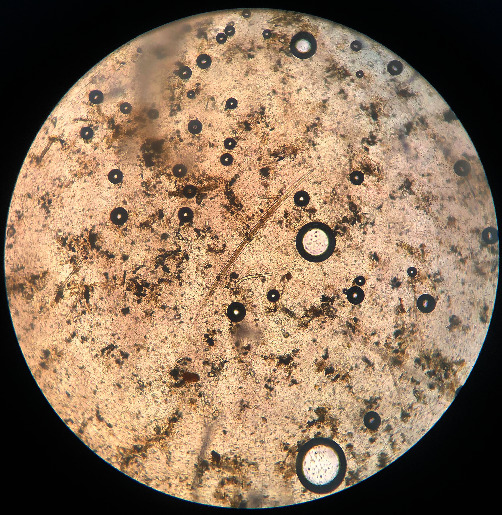
*Ostertagia*: head square, sheath tail forms a medium cone, and length 825–926 *μ*m. Third-stage larvae.

**Table 1:** The number (percentage) of the observed gastrointestinal parasites.

Genus	Spring	Summer	Fall	Winter	Total
*Nematodirus* ^a^	3 (5%)	0	2 (3.33%)	3 (5%)	8 (3.19%)
Trichuris^a^	2 (3.33%)	5 (8.33%)	5 (8.33%)	2 (3.33%)	14 (5.58%)
Toxocaridae^a^	2 (3.33%)	5 (8.33%)	5 (8.33%)	0	12 (4.78%)
*Strongyloides* ^a^	6 (10%)	0	0	0	6 (2.39%)
*Haemonchus* ^b^	4 (6.66%)	4 (6.66%)	3 (5%)	1 (1.66%)	12 (4.78%)
*Ostertagia* ^b^	3 (5%)	4 (6.66%)	7 (11.66%)	0	14 (5.58%)
Cooperia^b^	3 (5%)	3 (5%)	2 (3.33%)	0	8 (3.19%)
*Trichostrongylidae* ^b^	32 (53.3%)	33 (55%)	37 (61.6%)	15 (25%)	117 (46.61%)
*Oesophagostomum* ^b^	6 (10%)	4 (6.66%)	2 (3.33%)	0	12 (4.78%)
*Chabertia* ^b^	5 (8.33%)	3 (5%)	5 (8.33%)	2 (3.33%)	15 (5.98%)
*Dicrocoelium* ^a^	6 (10%)	2 (3.33%)	0	0	8 (3.19%)
*Fasciola* ^a^	9 (15%)	8 (13.33%)	8 (13.33%)	0	25 (9.96%)
Total	81 (32.27%)	71 (28.29%)	76 (30.28%)	23 (9.16%)	251

^a^Identified by its egg.

^b^Identified by its larvae.

**Table 2:** Comparison of the average percentage of sheep infected at different seasons of the year.

Season	Low infection intensity (%)	Moderate infection intensity (%)	High infection intensity	χ^2^
Spring	11.43	88.57	0	60.84^a^
Summer	12.5	87.5	0	56.63^a^
Fall	9.09	90.91	0	64.36^a^
Winter	88.89	11.11	0	60.84^a^
Total	30.47	67.27	0	60.66^a^

^a^A significant difference in the row (*P* < 0.01).

## Data Availability

Data supporting this research article are available from the corresponding author or first author on reasonable request.
